# The Potential Roles of Probiotics, Resistant Starch, and Resistant Proteins in Ameliorating Inflammation during Aging (Inflammaging)

**DOI:** 10.3390/nu14040747

**Published:** 2022-02-10

**Authors:** Dwina Juliana Warman, Huijuan Jia, Hisanori Kato

**Affiliations:** Health Nutrition, Department of Applied Biological Chemistry, Graduate School of Agricultural and Life Sciences, The University of Tokyo, 1-1-1 Yayoi, Bunkyo-ku, Tokyo 113-8657, Japan; warman-dwina-juliana681@g.ecc.u-tokyo.ac.jp (D.J.W.); akakeiken@g.ecc.u-tokyo.ac.jp (H.J.)

**Keywords:** gut health, inflammaging, probiotics, resistant starch, resistant proteins

## Abstract

Aging is typically accompanied by biological and physiological changes that alter cellular functions. Two of the most predominant phenomena in aging include chronic low-grade inflammation (inflammaging) and changes in the gut microbiota composition (dysbiosis). Although a direct causal relationship has not been established, many studies have reported significant reductions in inflammation during aging through well-maintained gut health and microbial balance. Prebiotics and probiotics are known to support gut health and can be easily incorporated into the daily diet. Unfortunately, few studies specifically focus on their significance in reducing inflammation during aging. Therefore, this review summarizes the scientific evidence of the potential roles of probiotics and two types of prebiotics, resistant starch and resistant proteins, in later age. Studies have demonstrated that the oral consumption of bacteria that may contribute to anti-inflammatory response, such as *Bifidobacterium* spp., *Akkermansia munichipilla*, and *Faecalis praunitzii*, contributes significantly to the suppression of pro-inflammatory markers in elderly humans and aged animals. Colonic fermentation of resistant starch and proteins also demonstrates anti-inflammatory activity owing to the production of butyrate and an improvement in the gut microbiota composition. Collectively, probiotics, resistant starch, and resistant proteins have the potential to promote healthy aging.

## 1. Introduction

Research interest in aging is gradually increasing, as the scientific community attempts to alleviate its detrimental effects by acquiring a more comprehensive understanding of this natural yet unstoppable process. As humans age, cellular functions decline, which leads to various physiological degradations, such as deteriorated cardiovascular structures and functions [1]; increased gut permeability, which increases susceptibility to pathogenic bacterial infections [2]; gastrointestinal tract disorders [3], which alter nutrition absorption and cause malnutrition in the elderly; degraded immune functions [4]; cognitive decline [5]; muscle frailty [6]; and gut dysbiosis [7]. Interestingly, the gut microbiota also coevolve as humans age [8]. Unfortunately, this leads to an imbalance in microbial composition and dominance of pathogenic and unbeneficial bacteria, a condition known as dysbiosis.

Some researchers postulate that dysbiosis also gives rise to chronic low-grade inflammation [7,9], which is linked to age-related chronic diseases, including cancer, dementia, and type 2 diabetes [8], while other researchers suggest that low-grade inflammation leads to dysbiosis [10,11]. Although the causal relationship between chronic low-grade inflammation and microbiota dysbiosis has yet to be definitively elucidated, the consensus is that they are strongly correlated as many studies have shown that a well-maintained gut health can reduce inflammation [7,12,13,14,15].

The gut microbiota composition is determined by many factors, with diet as the most predominant one. Our diet is the main source of nutrition, which is significant for daily bodily functions and the major contributing factor to gut microbiome composition and activity. A diet with low nutritional value tends to imbalance the gut microbial composition and trigger many health problems later in life.

Prebiotics are well established as one of the important substrates for gut microbial fermentation. Among the many sources of prebiotics, dietary fiber is considerably the most common. Resistant starch (RS), which has been classified as a new type of dietary fiber by the Food and Agriculture Organization (FAO), is characterized by a higher butyrate production from its fermentation compared with other dietary fibers [16,17,18,19,20]. Butyrate plays an essential role in providing energy to human colonocytes [21], preventing dysbiosis [22], and suppressing inflammation [23].

On the other hand, resistant proteins (RP), found in soybean, buckwheat, rice, potato, sericin, and eggshell membrane (ESM), also possess similar properties to RS but originate from proteins that are resistant to human proteases. Although they have not been scrutinized as widely as RS yet, several studies have reported their significant contributions to gut health [24,25,26]. Considering the limited reports on RP and gut health, this review is, to our knowledge, the first to highlight their prospects as elderly diet constituents to promote healthy aging.

In addition to prebiotics, probiotics (beneficial living bacteria) can be consumed orally. Some probiotics, such as *Lactobacillus acidophilus*, *Streptococcus thermophilus*, and *Bifidobacterium lactis*, are commonly known. Notably, *Bifidobacterium* spp., *Akkermansia muciniphila*, and *Faecalibacterium prausnitzii* also have high anti-inflammatory activities [27,28], and their oral intervention efficacies in the elderly have been reported.

As recently proclaimed by the United Nations (UN), 2021–2030 is the decade of healthy aging, highlighting the importance of well-being in later life [29]. By introducing and analyzing the potential roles of probiotics that may be involved in anti-inflammatory mechanisms, resistant starch, and resistant proteins in abating chronic low-grade inflammation that occurs in aging, this review is expected to contribute to the UN’s campaign on healthy aging.

## 2. Methods

For writing this narrative review, we performed a comprehensive search through PubMed and Google Scholar databases until December 2021. The keywords were “inflammation in aging”, “inflammaging”, “chronic low-grade inflammation”, “anti-inflammation” and “probiotics”, “resistant starch” and “aging”, “resistant starch” and “gut health”, “resistant protein” and “inflammation”, “resistant protein” and “gut health”. All in vivo studies in elderly and aged animals, review articles, and clinical studies reporting changes in inflammatory response (pro- and anti-inflammation cytokines) and gut microbiota composition were included. In addition, the World Health Organization or WHO’s website was also referred regarding the proclamation of the United Nations decade of healthy aging [29].

## 3. The Importance of Gut Health

Over the past decade, concerns regarding gut health have grown. Our intestinal tract, particularly the colon, is the dwelling site for both beneficial and pathogenic microbiota. Approximately 100 trillion microorganisms, mostly bacteria, are present in the human gastrointestinal tract [30]. They are considered master regulators of immune homeostasis, as their absence caused an impaired immune system [31]. While their role in immune system regulation is well established, they also protect the host from pathogens and unwanted microorganisms and produce thousands of metabolites that contribute to fitness and health [32].

The microbiota composition is regarded as a key factor in healthy gut physiology. According to Arumugam et al., the gut microbiota of adult humans is dominated by the phyla Firmicutes and Bacteroidetes, accounting for 90% of the total gut microbiota, with the rest being made up by other groups of bacteria with distinct functions and health benefits [33]. For instance, *Akkermansia muciniphila* was reported to protect human intestinal epithelial integrity via its outer membrane pili-like protein Amuc 1100 [34]. Studies in animals also showed that *A. municiphila* has notable anti-inflammatory activity in mice [28], as it supports the colonization of beneficial SCFA-producing bacteria in mice and macaque [35]. *Bifidobacterium* spp. generates lactate and acetate and reduces the population of inflammation-related microbes in human colon [36]. Toward et al. reported that the population of *Bifidobacterium* spp. was inversely correlated with the serum levels of inflammatory cytokines such as tumor necrosis factor alpha (TNF-α) and interleukin-1 beta (IL-1β) [37]. Butyrate-producing bacteria include *Clostridium* cluster XIVa, *Lachnospiraceae bacterium* [38], *Faecalibacterium prausnitzii* [27], *Coprococcus* spp. [39], and *Roseburia* spp. [39]. Among these, *F. prausnitzii* and *Roseburia intestinalis* have strong anti-inflammatory properties [27,40]. On the other hand, the abundance of some family of bacteria may be associated with health conditions, as lower population of *Christensenellaceae* family was reported in patients with Crohn’s disease [41,42,43,44], patients with ulcerative colitis [41,42], and people with body mass index (BMI) ≥30 [45].

In contrast, several pathogens and bacteria that may cause inflammation live in the gut, such as *Esterichia coli* [46], *Eggerthella lenta* [47], *Streptococcusgallolyticus* [48], and *Enterococcus* spp. [49]. An unhealthy diet and lifestyle significantly support the growth of these undesired bacteria, which suppresses the population of beneficial bacteria and increases the risk of various diseases. Hence, maintenance of the beneficial bacteria dominance in the gut is considered a strategy to sustain a healthy life considering their important roles and functions in the body (Table 1).

## 4. Aging-Related Changes: Gut Microbiota Composition and Chronic Low-Grade Inflammation

There is a considerable amount of literature published on the significant aging-related changes in gut microbiota composition, which greatly influences the general health of the host. Previously, the Western-style diet was the presumed cause of dysbiosis and the consequent aging-related inflammation [54]; in other words, external factors were thought to be the primary cause. However, Ragonnaud and Biragyn suggested recently that microbiota changes could be intrinsic to the aging process after their observations in Chinese and Italian elderly [8]. More interestingly, O’Toole and Jeffrey reported that despite the shift in gut microbiota balance, the core of microbiota did not age per se [55].

As indicated previously, several types of bacteria provide health benefits. Unfortunately, their population tends to decrease with age, and bacteria that promote chronic inflammation may replace them. The likely decrease or even disappearance of bifidobacteria with age has been well documented [56]. A study by Biagi et al. supported this by confirming the populations of *bifidobacteria*, some members of *Firmicutes*, including *Clostridium* clusters IV (*Ruminococcus obeum* et rel., *Roseburia intestinalis* et rel., *Eubacterium ventriosum* et rel., *E. rectale* et rel., and *E. hallii* et rel.), and some members of *Clostridium* cluster XIVa, including *Papillibacter cinnamovorans* et rel. and *F. prausnitzii* et rel., decreased in aged individuals and centenarians [12]. Typical beneficial bacteria, which are indicators of healthy aging, identified in Italian centenarians (99–104 years old) and semi-supercentenarians (105–109 years old) decreased during aging [57]. These included the *Ruminococcaceae*, *Lachnospiraceae*, and *Bacteridaceae* families.

**Figure 1:**
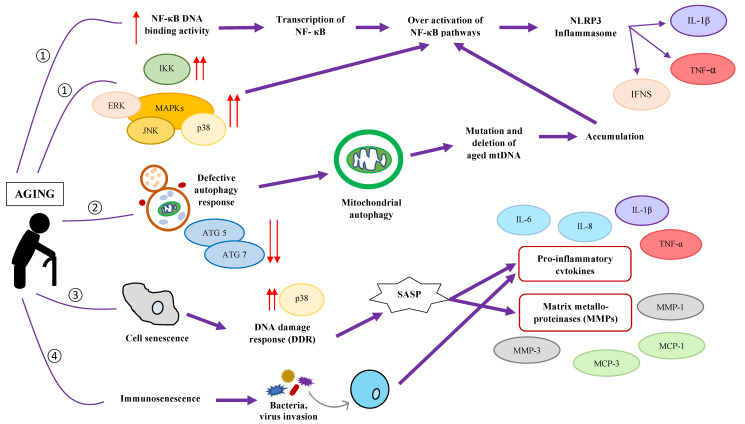
Illustration of the various mechanisms by which aging may activate inflammatory pathways [64]. An elevated NF-κB DNA binding activity increases the expression of the upstream kinases of NF-κB [65,66]. (2) Mitophagy and mtDNA mutation [67]. (3) Cell senescence activates DNA damage response (DDR) and senescence-associated secretory phenotype (SASP, which is a variation in phenotype induced by cell senescence) [68]. (4) Immunosenescence increases susceptibility of foreign materials invasion and activate the inflammatory response [69].

In addition to changes in gut microbiota composition, other physiological features also change significantly with age. Reduced organ functions due to aging, such as reduced cardiovascular structures and functions [1], increased gut permeability [2], gastrointestinal tract disorder [3], and degradation of immune function [4], have been widely reported. The degradation of immune function during aging is also termed immunosenescence and is suggested to be related to chronic low-grade inflammation or inflammaging. Chronic low-grade inflammation refers to an extended increase in pro-inflammatory factors at a level lower than typically observed in acute infections [58]. Figure 1 illustrates some mechanisms by which aging may contribute to chronic low-grade inflammation.

While studies on inflammation commonly use the pro-inflammatory cytokine interleukin-6 (IL-6) as a marker, the majority of studies investigating low-grade inflammation have utilized the surrogate marker high-sensitivity C-reactive protein (hsCRP) because of its extreme sensitivity to systemic inflammation [59,60,61]. Elevated IL-6 or hsCRP levels in the absence of an acute infection or other inflammatory stimuli indicate maladaptive chronic low-grade inflammation [62]. With advancing age, higher hsCRP and IL-6 levels are observed [61,63].

Chronic inflammation is a potential driver of metabolic disorders and age-related decline in physical functions [6], such as reduced mobility and impaired cognitive functions [5], and age-related diseases, such as cancer, depression, sarcopenia, and disability [70,71,72].

Many natural changes in the human body are hallmarks of aging, while chronic low-grade inflammation is one of the major contributing factors for various age-related diseases and disabilities in the elderly. Microbial composition dominated by bacteria that may promote inflammation also aggravates chronic inflammation, highlighting the profound effects of the gut microbiota on the health of the elderly.

## 5. How Gut Health Influences Inflammaging

The cause-and-effect relationship between inflammaging and gut health, that is, whether gut dysbiosis is the cause or consequence of inflammaging, remains unclear. Although there have been many studies, results are still inconclusive due to inconsistency. A study by Byndloss et al., for instance, suggested that the decrease in SCFAs altered the growth of beneficial bacteria and thus represented an opportunity for pathogens and bacteria that may induce inflammation to take over the gut colony [22]. In an aged mouse model, this microbiota transformation promoted dysbiosis and gut leakage, resulting in activated innate immune response or inflammation [9]. Age-related microbial dysbiosis is considered to contribute to systemic inflammation in humans [7]. A study by Nastasi et al. supported this finding, as the gut microbiota metabolites, particularly butyrate and propionate, reduced the pro-inflammatory chemokines by modulating the immune system in dendritic cells [73].

However, a contrasting result was described in a study by Thevaranjan et al. [11]. TNF-knockout old mice (>18 months old, equivalent to 60 years old human [74]) did not experience dysbiosis when the wild-type (WT) did, and the microbiome of old (but not young) mice changed after anti-TNF treatment. Similarly, Chen et al. indicated that dysbiosis occurred in mice that were deficient in the negative regulator of inflammatory signaling in a TNF- and IL-6-dependent manner [10]. Both studies proposed that inflammation triggered dysbiosis, notably in old mice.

Notwithstanding the cause-and-effect relationship between inflammaging and gut dysbiosis, the consensus is that they are intertwined. Although the causal relationship is complex, evidence supports the notion that healthy gut environment can reduce inflammation during aging.

## 6. Reducing Inflammaging by Improving Gut Health

**Figure 2:**
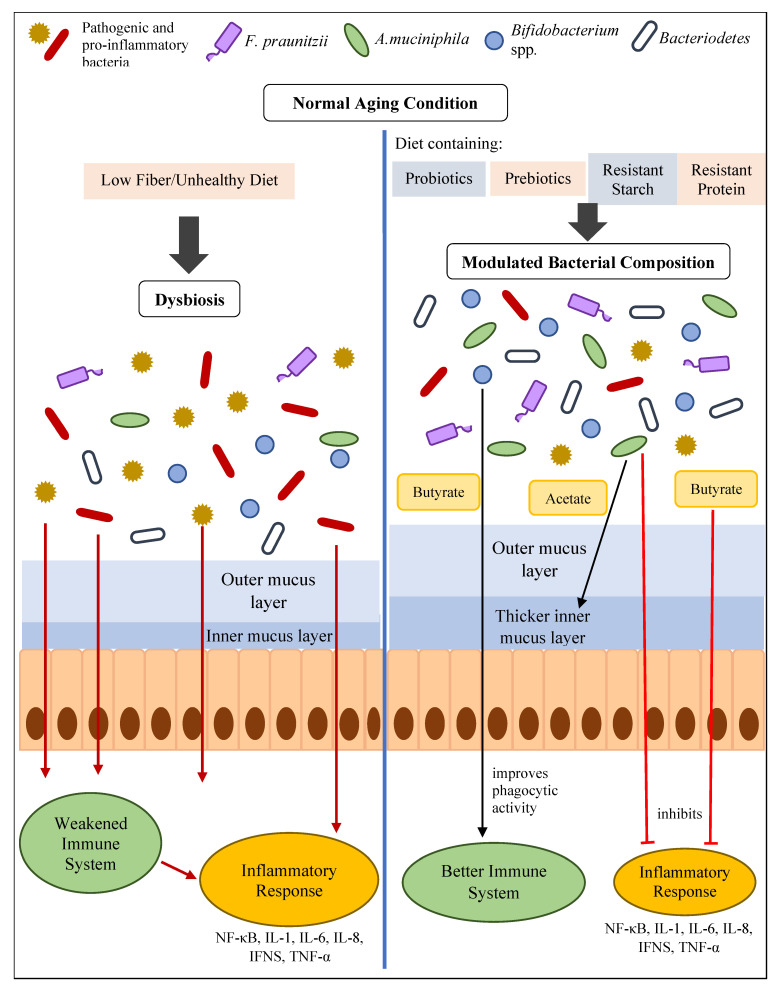
Illustration of the potential mechanisms by which probiotics, resistant starch, and resistant proteins contribute to the mitigation of aging-related chronic low-grade inflammation by producing SCFAs, improving phagocytic activity (*B. lactis*, *B. longum*), or directly reducing the production of pro-inflammatory cytokines.

There are multiple ways to improve gut health. Here, however, we focus on the consumption of butyrate-producing probiotics or probiotics with potential anti-inflammatory activities, resistant starch, and resistant proteins. Notably, their prospective impacts on aging-related chronic low-grade inflammation have been elaborated on. To the best of our knowledge, no previous review has addressed this topic. Based on this discussion, we expect to introduce a new approach to promote gut health through probiotics, resistant starch, and resistant proteins (Figure 2), which can be intensively investigated in the future.

### 6.1. Intervention of Probiotics with Potential Anti-Inflammatory Effects or Butyrate-Producing Probiotics

One type of beneficial metabolite produced by gut bacteria is short-chain fatty acids (SCFAs), which are produced by colonic fermentation of indigestible fibers [75]. The major SCFAs produced are acetate, propionate, and butyrate. Acetate is the most abundant and essential metabolite for the growth of other bacteria [52]. Butyrate mostly functions as the main energy source for human colonocytes [21], and it is essential for epithelial cells to prevent dysbiosis by maintaining the oxygen balance in the gut [22]. Liu et al. and Nastasi et al. also reported the anti-inflammatory properties of butyrate [23,73].

There is ample evidence that a high-fiber diet increases the production of SCFAs via microbial fermentation [76,77,78]. These organic acids were proven to improve survival against influenza in mice by inhibiting neutrophil recruitment and airway inflammation [50]. This may be explained by direct stimulation of immune cells, for example, by elevating both intestinal IgA and systemic IgG responses to curb pathogens [51].

SCFAs provide energy for beneficial bacteria, enterocytes, and immune cells. Ragonnaud and Biragyn stated that SCFAs could cross the gut epithelium layer to induce regulatory T cells (Tregs) and IL-10-producing T cells [8]. This activates anti-inflammatory responses in antigen-presenting cells and promotes the production of IgA and IgG by B cells, which increases immune tolerance.

As noted in the previous section, several species of bacteria with different functions are typically found in centenarians. In addition to bacteria that may attenuate inflammation, bacteria that produce SFCAs also contribute to healthy aging. Several recent reports revealed that the supplementation of some strains in the diet ameliorated age-related loss of mucin and demonstrated health and immune benefits in elderly and aged animals [28,79,80]. In addition to probiotic supplementation, fecal microbiota transplantation also effectively extends the lifespan of progeroid mice [81].

On the other hand, some probiotics, such as *Lactobacillus* spp. and bifidobacteria, are common supplements in dairy products or other functional foods. Detailed work by Maneerat et al. highlighted that the consumption of *Bifidobacterium lactis* Bi-07 could potentially strengthen the innate immune response in an elderly population without contributing to inflammation-related disorders [82]. The intervention by another species, *B. longum*, in healthy elderly people (65–90 years old) also contributed significantly to the suppression of inflammatory markers TNF-α and the increase in bifidobacteria (specifically *B. adolescentis*, *B. angulatum*, *B. bifidum*, and *B. longum*), fecal butyrate, and acetate concentrations [83]. The suppressing activities of *B. bifidum* BGN4 and *B. longum* BORI against bacteria with pro-inflammatory effects in the elderly (≥65 years old) led to the improvement of probiotic composition in the gut, as reported recently by Kim et al. [84].

*Akkermansia muciniphila* is another butyrate-producing bacterium with notable anti-inflammatory activities. Previous reports have established its anti-inflammatory activity in glucose-intolerant, obese, and aged animal models [79,85,86], as well as in humans [87,88]. In a study of accelerated aging Ercc1^−/Δ7^ mice, a decrease in mucus thickness was observed as an outcome of aging, whereas mice supplemented with *A. muciniphila* MucT (ATTC BAA-835) for 10 weeks (three times/week) showed a thicker layer due to the activation of colonic mucus production [28]. A thick mucus layer acts as a vital barrier against bacterial penetration, which causes inflammation. *A. muciniphila* supplementation also downregulated the expression of multiple genes and pathways related to inflammation and immune functions in both the colon and ileum. Ingenuine Pathway Analysis (IPA) predicted the inhibition of several inflammation-related factors upon supplementation, including pro-inflammatory IL-1, TNF receptor superfamily members 1B and 12, nuclear factor kappa B (NF-κB) inhibitor α, T-cell receptor (TCR), and Toll-like receptor adaptor molecule 1 (TICAM1). Although the anti-inflammatory properties of *A. muciniphila* have been extensively described previously [89], the study confirmed its protection against aging-related inflammation by reducing colonic expression of pro-inflammatory genes [28].

Like *A. muciniphila*, *Faecalibacterium praunitzii* is a butyrate-producing bacterium that may potentially contribute to the anti-inflammatory response. It mitigates inflammation in vitro and in vivo by blocking NF-κB activation and IL-8 production [90]. This is supported by significantly lower concentrations of IL-6, interferon gamma (IFN-γ), and IL-4 in the colon, and IL-6 and IL-22 in serum after 10-day supplementation in mice with dinitrobenzene sulfonic acid (DNBS)-induced chronic low-grade inflammation [91]. An association between this anti-inflammatory activity and butyrate production was hypothesized [91]. While the anti-inflammatory effects of *F. praunitzii* were widely reported previously, its protective activity against chronic low-grade inflammation was first revealed in mice by this seminal research.

However, the supplementation of probiotics may lead to safety issues, as not all bacteria have been clinically tested for oral consumption. Furthermore, some bacteria function in a diet-dependent manner, such as *Prevotella copri* [92,93], and the best way to enrich their colonization in the colon is by consuming more prebiotics. A study of gut microbiota composition in 168 long-lived Chinese (>90 years old) suggested alpha diversity, which depicts the abundance and evenness of species within a host [94], as a reasonable predictor of longevity [95]. This indicates that dietary and other interventions to maintain or promote diversity might be worth pursuing for healthy aging [7]. However, this view requires further scientific support. Another concern in probiotic supplementation is the viability of probiotics, which can survive in the human digestive system only long enough to reach the colon. Considering the above, incorporating prebiotics into our diets in addition to the oral consumption of probiotics may be a good strategy to increase gut microbiota diversity.

### 6.2. Prebiotics Intervention

Dietary fiber is widely regarded as an excellent source of prebiotics that can be naturally obtained from food, such as fruits, vegetables, whole grains, legumes, and nuts. Data from multiple sources have shown that a high-fiber diet is positively correlated with the enrichment of beneficial bacteria [96,97,98]. Similar to fermenting fiber, gastrointestinal microflora degrades other indigestible starch and protein fractions to produce SCFAs. These materials are referred to as resistant starches and resistant proteins. In recent years, research interests in resistant starch and resistant protein have been on the rise, and their potential benefits to human healthy aging have been demonstrated and warrant further discussion.

#### 6.2.1. Resistant Starch and Its Consumption in Elderly and Aged Animals

Most previous studies have defined resistant starch (RS) as a type of dietary fiber resistant to hydrolysis by human digestive enzymes. As it is indigestible, RS can reach the large intestine and is fermented by the gut microbiota to produce SCFAs. In other words, RS is a type of prebiotic. It is worth noting that the fermentation of resistant starch produces more butyrate than dietary fiber [16,17,18,19,20].

A recent in vitro study utilizing static batch fermentation with fecal slurry method re-confirmed that gut microbial fermentation of RS resulted in the highest accumulation of butyric acid compared to other dietary fibers [19]. This is because only RS enriches butyrate-producing bacteria that belong to *Bifidobacterium*, *Ruminococcus*, and *Roseburia* genera [19]. This is supported by research findings that *Ruminococcus bromii* and *Bifidobacterium* spp. degrade resistant starch more efficiently than other species [99,100,101]. Based on these findings and our focus on aging-related inflammation, we discuss resistant starch instead of other common prebiotics. The benefits of resistant starch in improving gut health have been reported, but to our knowledge, a review on its consumption and effects in the elderly has never been conducted previously.

RS is a common natural component of several types of food that can be categorized into four types based on its physical and chemical characteristics. RS1, generally found in whole grains and legumes, is an entrapped starch in a non-digestible matrix [102]. RS2 refers to ungelatinized starch granules, such as starch in raw potato and high-amylose cornstarch. The FDA has approved Hi-Maize resistant starch (naturally produced from modified high-amylose corn) for use in patients with type 2 diabetes [103]. RS3 consists of starch that has already undergone retrogradation (starch is cooled down after gelatinization). RS4, found in bread, includes starch that is chemically modified by adding ester or ether groups [102].

The correlation between RS consumption and gut health, inflammatory markers, insulin response, and lipid metabolism has been well documented. However, studies with elderly subjects are limited. We summarize relevant research findings here (Table 2) to provide an overview of the benefits of RS at later age.

RS should be considered for elderly diets because it can increase the population of beneficial bacteria and butyrate production according to multiple studies. In a serial study, MSPrebiotic^®^, a commercial RS containing 70% RS2 from *Solanum tuberosum* extract, promoted the growth of bifidobacteria and ameliorated dysbiosis related to the high abundance of Proteobacteria in subjects ≥70 years old [104]. Accordingly, changes in the levels of inflammatory markers (IL-10, C-reactive protein, and TNF-α) in blood were observed. Other investigations have shown that even though RS was able to reduce dysbiosis in the elderly, the inflammatory levels remained elevated at the end of the study [105]. It was hypothesized that three-month consumption of RS is not enough to alleviate the increased inflammatory response caused by colonocyte apoptosis, and earlier intervention of prebiotics in the diet (before 70 years of age) is required to prevent bowel damage, permeability, and the associated increase in non-specific inflammatory markers.

A similar result was found in a recent study using 18-month-old mice, reporting the therapeutic effects of RS2 against high-fat diet-induced and aging-related dysfunctions [103]. According to this study, RS2 effectively decreased the expression of systemic endotoxemia and pro-inflammatory cytokines, as evidenced by lower levels of serum and fecal lipopolysaccharides (LPS), which is an endotoxic component in the cell membrane of Gram-negative bacteria that induces inflammatory response, colonic IL-2, and hepatic IL-4. This corroborated the anti-inflammatory properties of RS2 against aging-related chronic low-grade inflammation. RS2 also enhanced gut barrier function, which was marked by increased expression of colonic mucin 2 at both the mRNA and protein levels. Moreover, this study revealed that RS2 reduced the abundance of pathogenic taxa associated with obesity, inflammation, and aging, such as *Desulfovibrio* (phylum Proteobacteria), *Ruminiclostridium* 9, *Lachnoclostridium*, *Helicobacteria*, *Oscillibacter*, *Alistipes*, *Peptococcus*, and *Rikenella* [103]. Collectively, studies both in humans and in mice support the view that RS2 can significantly improve gut health at a later age.

Changes in gut microbiota diversity in aged organisms are presumed to alter the microbial fermentation ability in the colon. To confirm this, Zhou et al. conducted a comprehensive study to examine RS tolerability in healthy aged mice (18 to 20 months old, analogous to 60–66 years old in humans [74]) and concluded that up to 36% of high-amylose-maize-resistant starch type 2 (HAMRS2) diets were well tolerated and, more importantly, could be fermented by aged mice in a dose-responsive manner as in young mice [106]. Furthermore, HAMRS2 increased colonic proglucagon expression and adiponectin levels in visceral fat, indicating improved insulin sensitivity in visceral fat. Increased cecal proglucagon expression is always linked to the elevation of circulating glucagon-like peptide 1 (GLP-1) in young rats, and GLP-1 can recuperate aging-related decline in glucose tolerance [109]. An identical outcome was established in a study conducted by Tachon et al. with the same mouse model [107]. They claimed that the improved microbiota composition (higher levels of Bacteroidetes, *Bifidobacterium* spp., *Akkermansia* spp., and *Allobaculum* spp.) in aged mice after 10-week supplementation with HAMRS2 was positively correlated with the expression levels of proglucagon.

Peixoto et al. investigated the effects of corn-based RS consumption in 11.5-year-old dogs [108]. The results showed that butyrate and total SCFA concentrations increased by approximately 49% and 36%, respectively, in the feces of dogs fed with high RS. Inflammation levels in the gut mucosa were also examined, but the differences were not significant. Nevertheless, this does not diminish the finding, as Swanson et al. pointed out that not all fermentable substrates, when included in diets, induce higher fecal butyrate levels [110]. This claim is supported by a study showing that the consumption of whole wheat-based RS infused into biscuits together with a canned diet did not elevate fecal butyrate levels in dogs [111].

Previous studies both in aged humans and in animals showed that RS consumption may contribute to the improvement of gut health and gut microbiota diversity; however, the results may depend on the host’s gut microbiota composition since it may vary between humans and between humans and animals. Moreover, individual intake should be monitored due to variance in intestinal physiology as the overconsumption of RS may cause diarrhea, vomiting, or soft stools. In other words, RS tolerance may vary among individuals. While some previous studies included tolerance tests for subjects, they were for reference only because the tolerance may vary drastically between animals and humans.

#### 6.2.2. Resistant Proteins

Resistant protein is not as well defined as resistant starch in the literature. In this review, resistant proteins (RP) refer to protein fractions indigestible by the human enzymatic digestive system that enter the colon and are fermented by the gut microbiota. They are different from undigested proteins, which refer to dietary proteins that escaped digestion or absorption in the small intestine. It is important to discern these two types of proteins because the metabolites and effects on the host are significantly different even though both are fermented in the colon. The fermentation of undigested dietary proteins primarily results in toxic products, such as ammonia, amines, phenols, and sulfides, which are associated with gastrointestinal tract diseases, particularly colorectal cancer and ulcerative colitis [112]. On the other hand, RP are fermented in the same manner as dietary fiber, which produces SCFAs and other metabolites derived from protein fractions that are indigestible by the human digestive system.

RP are found naturally in food, particularly plant-based foods, such as soybean, buckwheat, rice, and potato. Research on RP is scarce to date; thus the commonly known RPs are still limited to buckwheat protein, sericin, and the recently revealed eggshell membrane (ESM). Interest in RPs grew initially when the undigested high molecular fraction (HMF) of soy protein isolate (SPI) was found to be 25% less effective as a protein source compared to the SPI, and its consumption led to substantial bile acid binding by the post-digestive remnants of HMF and the subsequent elimination together with other excreta [113]. This bile acid-binding property is responsible for the hypocholesterolemic effect of RP, with a similar mechanism to that of dietary fiber and resistant starch.

Further investigation of RP led to the identification of sericin. Sericin is a natural macromolecular protein derived from the silkworm *Bombyx mori* and constitutes 15–35% of silk proteins [114]. Sasaki et al. reported that sericin, a silk protein, had low digestibility in the digestive system and a high water-holding capacity, and these properties led to the successful reduction of atropine-induced constipation in rats [26]. This suggested the effects of sericin, as a resistant protein, in mitigating constipation that were similar to those of dietary fiber.

According to Okazaki et al., sericin can be considered a prebiotic because it promotes colon health by modulating the immune response and intestinal barrier function [115]. They investigated the effects of sericin supplementation at 40 g/kg body weight in rats in a high-fat diet and observed elevated levels of fecal IgA and the presence of fecal mucins. As the gut barrier function weakens with age, sericin may be used to stimulate the immune system in the elderly. However, other typical prebiotic properties, such as promoting gut microbiota balance or acting as a substrate for beneficial microbial fermentation, have not been further investigated in sericin.

Recently, our research group identified eggshell membrane (ESM) as a resistant protein because of its low digestibility (approximately 46%) based on a study in rats [24]. ESM is a by-product of egg with interesting bioactivities, including anti-inflammatory activity, skin- and joint-health-promoting functions, and wound-healing properties [116,117,118]. We showed that ESM, as a resistant protein, could stimulate cecal fermentation and alter intestinal bacterial composition [24]. Dextran sodium sulphate (DSS)-induced IBD mice exhibited reduced richness in colon microbiota and dysbiosis. This condition, however, was alleviated by the ingestion of 8% ESM for 7 days. The populations of SCFA-producing bacteria, notably from the family of *Ruminococcaceae* and *Porphyromonadaceae*, which were principally decreased by intestinal inflammation, were restored. Considering the significant improvements in the gut environment after ESM intake, microbial diversity may be a major contributor to the anti-inflammatory activity of ESM.

Consistent with this finding, our research further confirmed that ESM could improve the inflammatory response by modulating gut microbiota composition [25]. Supplementation with 8% ESM in high-fat diet (HFD)-fed mice significantly suppressed the proliferation of inflammation-related bacteria (*Roseburia faecis*, *Ruminococcus callidus*, and *Blautia hydrogenotrophica*) and obesity-related bacteria (*Blautia coccoides* and *Parabacteroides goldsteinii*) at 20 weeks. Although the concentrations of cecal acetate, lactate, butyrate, and propionate were not affected by either the high-fat or the ESM diet, cecal isobutyrate levels were higher in the ESM group than in the HFD and control groups. The negative correlations between the abundance of *P. goldsteinii* and *B. hydrogenotrophica* and isobutyrate concentration suggest that high isobutyrate levels are unfavorable to the obesity-related bacteria, which is consistent with previous findings by Wang et al. [119]. Collectively, these studies provide important evidence that ESM, as a novel source of resistant proteins, has the potential to reduce dysbiosis, which is a determining factor for healthy aging. In the future, we are interested in investigating the anti-inflammatory effectiveness of ESM against aging-related chronic low-grade inflammation.

## 7. Future Studies

In studies using aged humans and animals, probiotics, resistant starch, and resistant proteins show potential effects in promoting gut health and mitigating inflammation in aging. However, there are differences in the gut microbiota composition between humans and animals. Even between humans, the gut microbiota may be influenced by gender, lifestyle, and geographical factors. Therefore, studies investigating the role of probiotics, resistant starch, and resistant proteins by considering these factors can be addressed in the future. The findings in animal experiments we have summarized in this review can be a basis for further clinical studies to provide more empirical data. Underscoring the prospective role of resistant protein as a prebiotic, we have interest in continuing our work on ESMs. Future studies may investigate the mechanism of how ESMs may improve gut microbiota composition or how they may contribute to the production of beneficial metabolites, such as short-chain fatty acids. Considering its potential anti-inflammatory activity, the relationship between ESM intake and immune system, particularly in relation to aging to promote healthy aging, is worth a further investigation.

## 8. Conclusions

Multiple studies have demonstrated that typical probiotics possessing anti-inflammatory activities and resistant starch can abate aging-related chronic low-grade inflammation in humans and animal models. The main mechanisms include the direct increase of bacterial population, particularly bifidobacteria, *A. muciniphila*, and *F. prautnitzii*, and the supply of substrates for colonic microbial fermentation in the form of indigestible starch. Besides the efficacy, the dosage and consumption frequency of resistant starch should be monitored, particularly for individuals with sensitive digestive tracts or low tolerance to dietary fiber. On the other hand, despite limited studies on resistant proteins and their interventions in the elderly, recent studies have disseminated their fermentabilities to produce SCFAs and promising potential in ameliorating gut dysbiosis and thus their consequent protection against inflammation. All findings elaborated in this review are expected to trigger further exploration of resistant proteins, probiotics, and resistant starch in maintaining gut health, targeting inflammaging, and promoting healthy aging.

## Figures and Tables

**Table 1:** The importance of several bacteria and their fermentation products to the host.

Component	Role or Function
Microbial Fermentation Metabolites	
SCFAs (overall)	Stimulating immune system [50] Providing energy for and supporting the growth of beneficial bacteria [8] Increasing both intestinal immunoglobulin A (IgA) and systemic immunoglobulin G (IgG) responses to prevent pathogens growth [51]
Acetate	Supporting the growth of probiotics [52]
Butyrate	Primary energy source for human colonocytes [21] Preventing dysbiosis in epithelial cells [22] Reducing pro-inflammatory cytokines and chemokines [23]
Propionate	Lowering lipogenesis, serum cholesterol levels, and carcinogenesis in other tissues [53]
Probiotics	
*Christensenellaceae* family	Associated with health conditions, particularly body mass index (BMI) and inflammatory bowel disease (IBD) [41,42,43,44,45]
*Akkermansia* *muciniphila*	Anti-inflammatory activities [28] Protecting intestinal epithelial integrity [34] Supporting colonization of SCFA-producing bacteria [35]
*Bifidobacterium* spp.	Producing lactate and acetate that can reduce the population of pathogenic bacteria [36] Suppressing serum levels of pro-inflammatory cytokines [36]
*Clostridium* cluster XIVa	Producing butyrate [38]
*Lachnospiraceae* *bacterium*	Producing butyrate [38]
*Faecalibacterium prausnitzii*	Producing butyrate [27] Strong anti-inflammatory properties [27]
*Coprococcus* spp.	Producing butyrate [39]
*Roseburia* spp.	Producing butyrate [39] Strong anti-inflammatory properties [40]

SCFAs, short chain fatty acids.

**Table 2:** Summary of study investigating the effects of resistant starch intake in either aged human or animal subjects.

Reference	Subject	Type of Resistant Starch	Methods	Effects
[104]	Elderly (≥70 years old) compared to middle-aged adults (30–50 years old)	MSPrebiotic^®^ **	Prospective, placebo-controlled, randomized, double-blinded study, 30 g of RS intervention for 3 months.	↑ bifidobacteria ↓ Proteobacteria dysbiosis
[105]	Elderly (≥70 years old)	MSPrebiotic^®^ **	Prospective, blinded, placebo-controlled study, 30 g of RS consumption for 12 weeks.	↓ blood glucose and insulin resistance (↓ type-2 diabetes risk in elderly)
[103]	Healthy 18-month-old mice *	HAMRS2	High-fat diet, supplemented with 20% of RS2 for 16 weeks.	↓ systemic endotoxemia expression, pro-inflammatory cytokines (LPS, IL-2, IL-4) ↑ gut barrier function ↓ pathogen related to obesity, inflammation, and aging
[106]	Healthy 18 to 20-month-old mice *	HAMRS2	0, 18, and 36% of HAMRS2 diet for 10 weeks.	↑ gut microbial fermentation ↑ cecal proglucagon (recuperate aging-related decline in glucose tolerance)
[107]	18 to 20-month-old mice *	HAMRS2	0, 18, and 36% of HAMRS2 intervention for 10 weeks.	↑ Bacteroidetes, *Bifidobacterium* spp., *Akkermansia* spp., and *Allobaculum* spp. ↑ proglucagon level
[108]	11.5-year-old dogs	RS	Feed added with 1.46% RS was given for 51 days.	↑ fecal butyrate and total SCFA concentrations

* Mice 18 to 20 months of age are equivalent to 60–66-year-old humans [74]. ** MSPrebiotic^®^ is a commercial resistant starch product containing 70% RS2 from *Solanum tuberosum* extract [105]. HAMRS2: high amylose maize resistant starch type 2; RS: resistant starch; LPS: lipopolysaccharide; SCFA: short-chain fatty acid. ↑: increase, ↓: reduce or decrease.

## Data Availability

Not applicable.
